# High-gain polymer optical waveguide amplifiers based on core-shell NaYF_4_/NaLuF_4_: Yb^3+^, Er^3+^ NPs-PMMA covalent-linking nanocomposites

**DOI:** 10.1038/srep36729

**Published:** 2016-11-09

**Authors:** Meiling Zhang, Weiwei Zhang, Fei Wang, Dan Zhao, Chunyang Qu, Xibin Wang, Yunji Yi, Eric Cassan, Daming Zhang

**Affiliations:** 1 State Key Laboratory on Integrated Optoelectronics, College of Electronic Science and Engineering, Jilin University, Changchun, 130012, China; 2 Centre for Nanoscience and Nanotechnology (C2N, Orsay), Université Paris Sud, CNRS, UMR 8622, Université Paris-Saclay, Bât. 220, 91405 Orsay Cedex, France

## Abstract

Waveguide amplifiers have always been significant key components for optical communication. Unfortunately, the low concentration of rare earth ions doped in the host material and the inadequate optimization of the waveguide structure have been the common bottleneck limitations. Here, a novel material, NaYF_4_/NaLuF_4_: 20% Yb^3+^, 2% Er^3+^ nanoparticle-Polymeric Methyl Methacrylate covalent-linking nanocomposite, was synthesized. The concentrations of Er^3+^ and Yb^3+^ doping increased an order of magnitude. Under a 980 nm laser excitation, highly efficient emission at 1.53 μm was obtained. The characteristic parameters of the single mode waveguide were carefully designed and optimized by using a finite difference method. A formulized iteration method is presented for solving the rate equations and the propagation equations of the EYCDWA, and both the steady state behavior and the gain were numerically simulated. The optimal Er^3+^ and Yb^3+^ concentrations are 2.8 × 10^26^ m^−3^ and 2.8 × 10^27^ m^−3^, and the optimal waveguide length is 1.3 cm. Both theoretical and experimental results indicated that, for an input signal power of 0.1 mW and a pump power of 400 mW, a net gain of 15.1 dB at 1530 nm is demonstrated. This result is the highest gain ever reported in polymer-based waveguide amplifiers doped with inorganic Er^3+^-Yb^3+^ codoped nanocrystals.

In the past two decades, erbium-doped fiber amplifiers have been extraordinarily successful because the infrared emission wavelength of the Er^3+^ ion corresponds to the low-loss telecommunication window in the wavelength range approximately 1530 nm in optical communication^1^,^2^,^3^. With continued progress in information technology, the miniaturization of such fiber amplifiers is of considerable interest for the development of on-chip photonic integration and chip-scale optical communication. The continuous decrease in both size and cost has provided a motivation for utilizing erbium-doped waveguide amplifiers (EDWAs)^4^. EDWAs can be integrated with other optical devices, such as splitters, couplers, and switches as active integrated optical waveguide devices networks, which have important applications in optical communication, phase array antennas, optical computers, and optical systems. Erbium-doped waveguide amplifiers could offer a unique highly integrated optical signal processing platform with benefits of low loss, compact, flexible design of optical properties, and cost-effective features for high density integrated devices^5^,^6^. Various materials have been investigated to fabricated EDWAs, including Er^3+^ doped in Al_2_O_3_^7^,^8^, Y_2_O_3_, LiNbO_3_ crystal^9^,^10^, phosphate glass^11^, and polymeric materials^12^. Compared with the other hosts, organic polymers are exemplary, with their excellent properties in terms of simple processing, low-cost, high bandwidth, and good thermal stability^13^,^14^.

EDWAs have recently received considerable attention as a potential high-gain medium for optical amplification in the communication band. To increase the absorption cross section, ytterbium ions (Yb^3+^) are usually codoped as a sensitizer. Er^3+^-Yb^3+^ codoped waveguide amplifiers (EYCDWAs) are therefore expected to be an attractive high-gain medium material for optical amplification^15^,^16^,^17^,^18^. With the development of optical communication, optical waveguide amplifiers with high gain are required urgently. In particular, according to the current reports, developments in the net gain are expected. Anh Quoc Le Quang *et al.* fabricated an EDWA based on erbium-complex-doped Polymeric Methyl Methacrylate (PMMA) thin films and demonstrated a net gain of 1.34 dB at 1540 nm for a 1.6 cm long single mode waveguide amplifier^19^. Er^3+^-Yb^3+^ doped ENR polymer optical amplifiers with optical gains approaching 4.85 dB/cm^−1^ were also reported^20^. Obviously, such characteristics do not meet the modern optical communications requirements; thus, the current efforts in improving the gain properties of the EYCDWAs are not sufficient. The low concentration and poor dispersion of the inorganic Er^3+^ and Yb^3+^ ions doped directly in organic polymeric matrices have become the bottleneck for progress. Simultaneously, more attention should be paid to optimize the device structures. In 2015, Tianjiao Wang *et al.* synthesized NaYF_4_:Er^3+^, Yb^3+^ NPs-PMMA covalent-linking nanocomposites with core only using a high temperature thermal decomposition approach, and fabricated the waveguide amplifiers. A relative optical gain of 7.6 dB was obtained at 1540 nm in a 15 mm-long waveguide^21^. In this case, the doping mass concentration of NPs in the polymer matrix could be up to 1%, which is larger than previously published results. While their luminescence quantum yield was generally low due to nonradiative energy losses caused by the surface defects as well as from vibrational deactivation ascribed to solvent molecules and ligands adsorbed on the NPs. To overcome this deficiency, one effective strategy to improve the luminescence of NPs is the construction of core–shell architectures, where a shell is grown around the luminescent core with similar lattice constants.

In this paper, we report a new strategy to improve the dispersibility of nanoparticles (NPs) in a polymer matrix, in which the core-shell NaYF_4_/NaLuF_4_: Yb^3+^, Er^3+^ NPs were copolymerized with methyl methacrylate (MMA) and NPs-PMMA covalent-linking nanocomposites were synthesized. Using the NPs-PMMA covalent-linking nanocomposites as the core material, we constructed optical waveguide amplifiers with a structure of an embedded waveguide. A formulized iteration method is presented for solving the rate equations and the propagation equations of the EYCDWA under the conditions of uniform doping and both the steady state and the gain were numerically simulated. The characteristic parameters were optimized. The device was fabricated and characterized using the conventional semiconductor processes. A comparison of the experimental results with the theoretical model is presented. Both simulation and analysis were performed for this material. For an input signal power of 0.1 mW and a pump power of 400 mW, a net gain of 15.1 dB at 1530 nm was demonstrated for waveguide lengths of 1.3 cm; the concentration of Er^3+^ was 2.8 × 10^26^ m^−3^ and the concentration of Yb^3+^ was 2.8 × 10^27^ m^−3^. This net gain result is the highest ever reported in polymer-based EYCDWAs containing erbium doped inorganic nanocrystals.

## Results and Discussion

### Structure Design

In our experiment, a novel type of active material, NaYF_4_/NaLuF_4_: Yb^3+^, Er^3+^ NPs-PMMA, was synthesized. The core-shell NaYF_4_/NaLuF_4_: Yb^3+^, Er^3+^ NPs has not only improved the intensity of the 1.53 µm fluorescent band but also suppressed surface quenching. NaYF_4_/NaLuF_4_: Yb^3+^, Er^3+^ coated with oleic acid (OA) was synthesized using a mild hydrothermal method in a water-ethanol-OA mixed solvent system. The surface of NPs was modified by the unsaturated functional groups. Next, these NaYF_4_/NaLuF_4_: Er, Yb NPs were copolymerized with methyl methacrylate (MMA), and then the NPs-PMMA covalent-linking nanocomposites were synthesized. Distinguished from the physical doping, this novel material has a higher concentration of dopant and its performance is more stable. Under 980-nm laser excitation, highly efficient emission at 1.53 μm was obtained.

**Figure 1:**
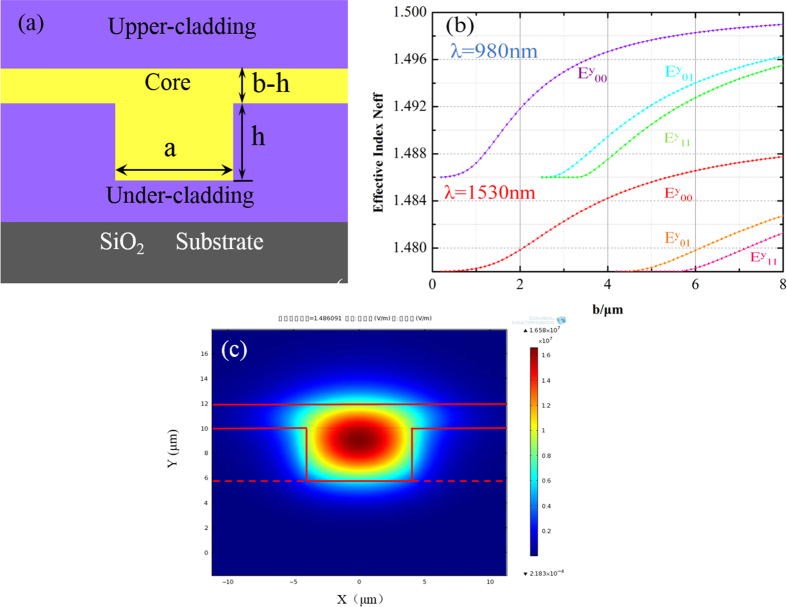
Characterization of the rib waveguide. (**a**) The cross section of the rib waveguides; (**b**) relationships between core thickness *b* and the effective refractive indices *N*eff of the rib waveguide with *a* = 1.5*b* and *h* = 0.7*b*; (**c**) the optical field distribution of the signal in a rib waveguide at 1530 nm.

Taking into account the high concentrations of dopant in the NaYF_4_/NaLuF_4_:Yb^3+^, Er^3+^ NPs-PMMA films, it is very difficult to directly etch the film to form rib or rectangular waveguides. Therefore, we choose the embedded waveguide as the structure. The cross-section view of the waveguide is shown in Fig. 1(a). The NaYF_4_/NaLuF_4_: Yb^3+^, Er^3+^ NPs-PMMA was used as a core, and the PMMA was used as a cladding. The refractive index of the core and upper cladding were measured using an ellipsometry method (J. A. Woollam., Co.M2000). The measured values of the core layer were 1.49 and 1.5001 at 1530 nm and 980 nm, respectively. The refractive indices of the cladding were 1.478 and 1.486 at 1530 nm and 980 nm, respectively. The dimension of the waveguide and the refractive index of the core layer and cladding often determine the single-mode confinement condition. Signal-mode field^22^ simulations at the pump and signal wavelengths were conducted using the finite difference method (FDM). The relations based on the eigenvalue equations between the core thickness band mode effective refractive indices *Neff* of the rib waveguide are shown in Fig. 1(b), where the waveguide width *a* = 1.5*b* and the rib height *h* = 0.7*b*. As visible, we can realize single-mode propagation of the mode when *b* was between 0 and 3 μm at 980 nm and 0 to 5 μm at 1530 nm realize in the rib waveguide. *b* = 5 μm (*a* = 7.5 μm and *h* = 3.5 μm) was chosen as the total core thickness. Figure 1(c) shows the optical field distribution at 1530 nm of this size of waveguide calculated using the beam propagation method (BPM).

### Gain characteristics

**Figure 2:**
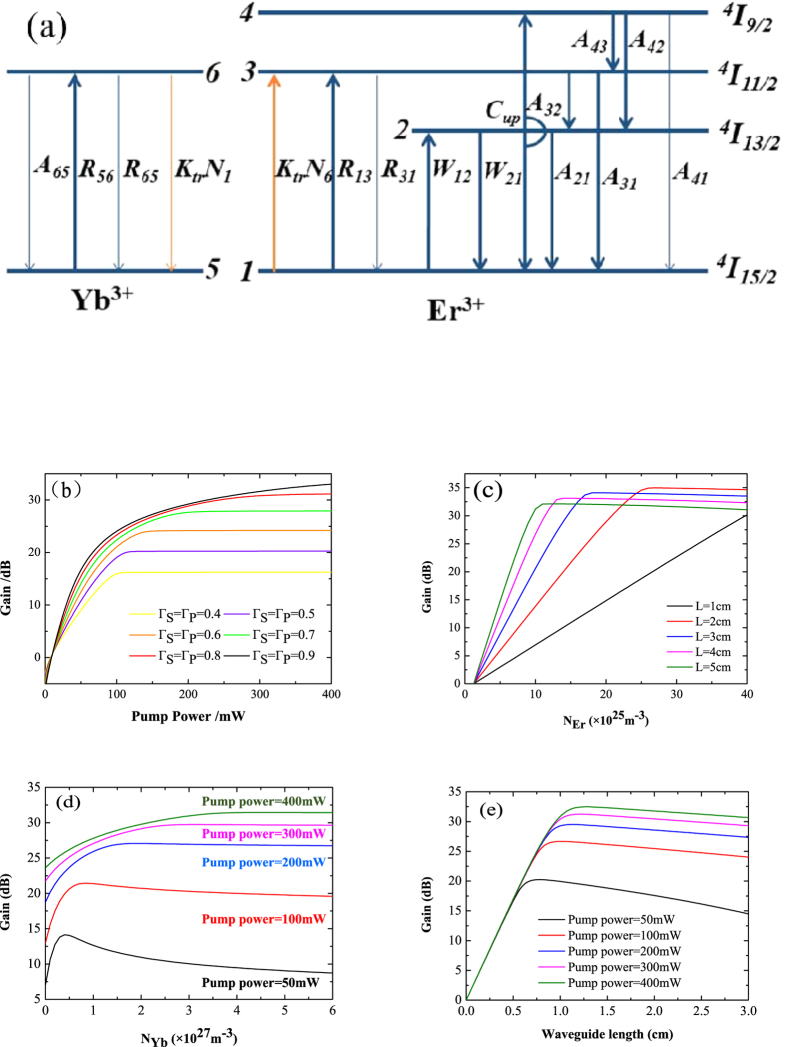
Analysis of the gain characteristics. (**a**) Energy level transitions for Er^3+^–Yb^3+^ co-doped systems. (**b**) The gain versus coordinate pump power at 980 nm for different overlapping factors. (**c**) The gain versus Er^3+^ concentration *N*_Er_ for different waveguide length *L*. (**d**) The gain versus Yb^3+^ concentration *N*_Yb_ for different pump powers. **(e)** The gain versus waveguide length for different pump powers.

Optimization of the parameters of the waveguide is very important. To achieve a more accurate simulation of the gain characteristics, it is necessary to consider as many levels as possible of the Er^3+^–Yb^3+^ co-doped systems shown in Fig. 2(a). The upconversion effect between erbium ions and the cross relaxation effect between erbium-ytterbium ions were considered. The operation principle of the EYCDWA is described as follows. Yb^3+^ ions are excited by the pump light from the ground state ^2^F_7*/*2_ to the excited state ^2^F_5*/*2_, followed by the transfer energy to the nearby ground state Er^3+^ ions ^4^I_15/2_. Next, these Er^3+^ ions are excited to the excitation level ^4^I_11/2_. Because the excitation level ^4^I_11/2_ is unstable, Er^3+^ ions quickly decay to the metastable level ^4^I_13/2_. Meanwhile, upconversion occurs between two Er^3+^ ions from the signal level ^4^I_13*/*2_ to both ^4^I_9*/*2_ and ^4^I_15*/*2_. Population inversion occurs from ^4^I_13*/*2_ to ^4^I_15*/*2_, followed by relaxation to the ground level ^4^I_15/2_ via emission of photons, the frequency of which is the same as that of the signal; thus, the device achieves amplification of the signal. Here, the amplified spontaneous emission was neglected. Therefore, the rate equations were established and then solved using a formulized iteration method. Combining the signal and the pump propagation equations, the gain characteristics were analyzed using the Runge-Kutta method under the conditions of the uniform doping and the steady state. The gain *G* was defined as follows^23^,^24^:
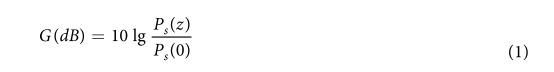


The gain curves were simulated accordingly. Figure 2(b) shows the gain versus coordinate pump power at 980 nm for different overlapping factors. The overlap integral factor plays a decisive role on the gain. The formula is given as follows^25^:
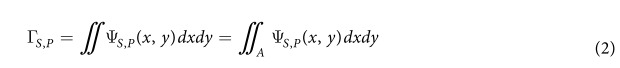
where *Γ*_*P*_ and *Γ*_*S*_ are the overlap factors of the pump and the signal, respectively. *A* is the area of the cross-section of the waveguide core. Figure 2(b) shows that as the overlap integral factors increase, the gain increases and then saturates at the pump power of 400 mW. When the overlap factors change from 0.8 to 0.9, the gain does not change significantly. The overlap factors of the embedded waveguide with the size we designed are 0.848 at the signal wavelength and 0.87 at the pump wavelength. The overlap factors change to different waveguide cross-section dimensions. The simulation results shown above indicate that the gain highly depends on the overlapping factors related to the waveguide cross-section dimensions. The results provide further verification that the dimensions of the waveguide cross-section we designed are valid and that the pump power is sufficient.

Based on the above analysis, we choose 400 mW as the pump power because the gain curve tends to saturation around this level. Thus, the erbium ion concentration and the waveguide length are optimized. Figure 2(c) shows the gain versus Er^3+^ concentration *N*_Er_ for different waveguide lengths. Figure 2(c) indicates that as the Er^3+^ concentration increases, the gain increases to a maximum. The Er^3+^ concentration corresponding to the maximum gain is called the optimal Er^3+^ concentration, which has different values under different waveguide lengths. When the length of the waveguide increases from 1 cm to 4 cm, the gain corresponding to the optimal Er^3+^ concentration also increases. Moreover, for the length of the waveguide of 5 cm, the gain decreases under the pump power of 400 mW. Therefore, to obtain high gain, the Er^3+^ concentration cannot be excessively high. Its, value can be chosen in the range of 1.6–2.8 × 10^26^ m^−3^ for the waveguide length of 1–2 cm. The optimal Er^3+^ concentration is 2.8 × 10^26 ^m^−3^ with waveguide length of approximately 2 cm. The gain of which is 35 dB. Ytterbium ions codoped as a sensitizer can increase the concentration of Er^3+^ to prevent quenching. Thus, the Yb^3+^ concentration has a substantial impact on the gain. For the pump power of 400 mW, the Er^3+^ concentration is 2.8 × 10^26^ m^−3^ and the waveguide length is 2 cm, and then the Yb^3+^ concentration is optimized. The gain versus Yb^3+^ concentration *N*_Yb_ curves for different pump powers are shown in Fig. 2(d). Figure 2(d) indicates that the gain increases with the pump power increasing. As the Yb^3+^ concentration increases, the gain increases to a maximum and then decreases. The optimal Yb^3+^ concentration is 2.8 × 10^27^ m^−3^ under the 400 mW pump power. From the above analysis we obtained the optimized waveguide range of 1–2 cm; to obtain an optimized value of the waveguide length accurately, the gain versus waveguide length for different pump powers were simulated, as shown in Fig. 2(e). We know from Fig. 2(e) that as the pump power increases, the maximum gain also increases. As the waveguide length increases, the gain increases to a maximum and then decreases. The waveguide length corresponding to the maximum gain is called the optimal waveguide length, which has different values for different pump powers. When the pump power is 400 mW, the optimal waveguide length is 1.3 cm. All the optimal parameters from the above analysis are summarized in Table 1.

### Comparison of the experimental results with the simulation

**Figure 3:**
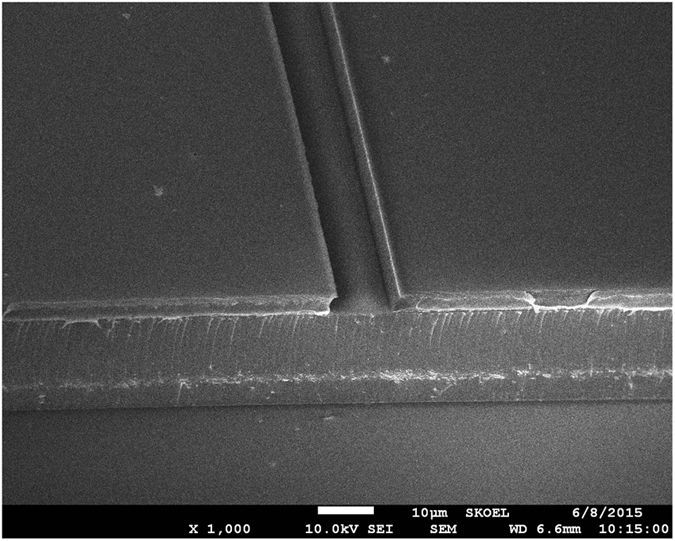
The SEM micrograph of the groove cross-section without embedding the NaYF_4_/NaLuF_4_: Yb^3+^, Er^3+^ NPs-PMMA.

The NaYF_4_/NaLuF_4_:Yb^3+^, Er^3+^ NPs-PMMA was used as a core, and the PMMA was used as the cladding. The dimensions of the waveguide were determined by the above simulation. The width of the groove is 7.5 μm, and the depth is 3.5 μm. We used conventional semiconductor processes to fabricate the embedded waveguide. Figure 3 shows the scanning electron microscopy (SEM) micrograph of the cross-section of a groove. The ridge wall is smooth and almost vertical. The width of the groove is 7.7 μm, and the depth of the groove is 3.8 μm. These dimensions are basically consistent with the dimensions we designed. The thickness of the upper cladding and the under cladding is 3 μm and 7 μm, respectively.

**Figure 4:**
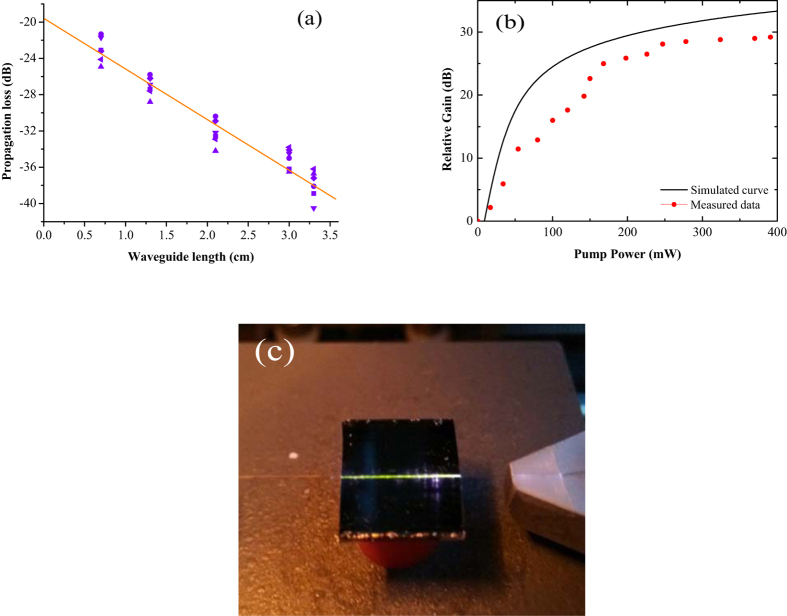
Characteristic of the waveguide amplifier. (**a**) The propagation losses at 1530 nm of the waveguides with different lengths. (**b**) The measured and the simulated relative gain as a function of pump power at 980 nm in a 1.3-cm long waveguide. (**c**) The tested sample of the waveguide amplifier with upconversion luminescence under 980-nm laser excitation.

The propagation losses of the waveguide amplifier were measured by the cut-back method^26^. The cutback method relies on assuming identical fiber-to-waveguide coupling conditions and comparing the losses at1530 nm through waveguides of different lengths in the same waveguide, as shown integrated radially in Fig. 4(a). A linear fit to the experimental data is shown as a continuous line, and its slope provides the propagation-loss coefficient in dB/cm. The propagation loss is approximately 5.3 ± 0.3 dB/cm at 1530 nm. This value is relatively high because the absorption cross-section of the Er^3+^ is high, and the corresponding strong absorption was considered in the simulation. The gain characteristics of the EYCDWA were tested. Figure 4(b) shows the measured data and the simulated curve as a function of pump power at 980 nm in a 1.3-cm long waveguide. The solid line shows the simulated results based on the theoretical models, and the solid square dots are the measured data. When the input signal power was 0.1 mW and the pump power was 400 mW, a high relative gain of 29.2 dB at 1530 nm was achieved. Compared with the simulated result, the measured data were close to the theoretical estimates. Figure 4(c) shows the tested sample of waveguide amplifiers for the optical gain measurement. Under 980-nm laser excitation, a clear green light from the phenomenon of upconversion occurs, which we have considered the upconversion in our above models. Next, the total insertion losses were measured to estimate the coupling loss immediately after each gain measurement. Because the launching and collecting fibers have exactly the same characteristics for the gain measurements and index-matching-fluid (diphenyl ether) was used in between the fiber and waveguide facets during all the experiments, it was assumed that similar coupling efficiencies were achieved in both the input and output ports. The coupling loss calculated by subtracting the previously determined propagation loss from the total insertion loss was approximately 3.6 dB per facet. Subtracting the propagation loss and the coupling loss, a net gain of 15.1 dB was achieved in a 1.3-cm long waveguide. Polymer optical waveguide amplifiers based on NaYF_4_/NaLuF_4_: 20% Yb^3+^, 2% Er^3+^ NPs-PMMA covalent-linking nanocomposites with 15.1 dB net gain is the highest net gain ever reported for polymer optical waveguide amplifiers doped with nanocrystals. But there is still a difference between the obtained results and the simulated amplifier performance. One of the reasons was the large coupling loss of the end faces. The large coupling loss could lead to the small power of the signal light and pump light coupled into the waveguide. Even though the coupling loss was subtracted in the simulated results, the small power of the signal light and pump light could decreased the gain. Besides, the ridge wall in simulation was more smooth and vertical than the waveguide fabricated. Then the propagation loss of the waveguide was larger than theoretical simulation. In addition, the fabrication tolerances can also result in the difference.

## Conclusion

In conclusion, we designed an EDWA with a structure of an embedded waveguide using the NaYF_4_/NaLuF_4_: 20% Yb^3+^, 2% Er^3+^ NPs-PMMA covalent-linking nanocomposite as the core material. The concentrations of Er^3+^ and Yb^3+^ doping increased by an order of magnitude. Under 980-nm laser excitation, highly efficient emission at 1.53 μm was obtained. The characteristic parameters of the single mode waveguide were carefully designed and optimized using a finite difference method. A formulized iteration method was presented for solving the rate equations and the propagation equations of the EYCDWA under the conditions of uniform doping, and both the steady state behavior and the gain were numerically simulated. The optimal Er^3+^ and Yb^3+^ concentration is 2.8 × 10^26^ m^−3^ and 2.8 × 10^27^ m^−3^, respectively, and the optimal waveguide length is 1.3 cm. Both theoretical and experimental results indicated that, for an input signal power of 0.1 mW and a pump power of 400 mW, a net gain of 15.1 dB at 1530 nm is demonstrated for waveguide lengths of 1.3 cm. This result is the highest net gain ever reported in polymer-based on Er^3+^-Yb^3+^ codoped waveguide amplifiers doped inorganic nanocrystals.

## Methods

### Sample fabrication

**Figure 5:**
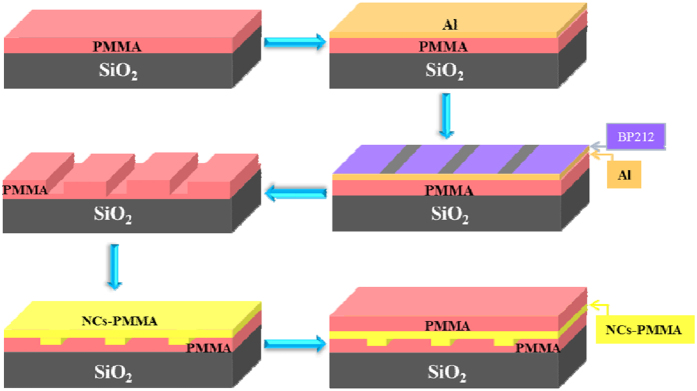
Fabrication processes for polymer optical waveguide amplifiers.

We use the conventional semiconductor processes of spin-coating, standard photolithography and inductively coupled plasma (ICP) etching technology to fabricate the EYCDWA. Figure 5 shows a complete fabrication process diagram. A 7-μm-thick PMMA film was first spin-coated onto a 2-μm-thick silicon dioxide layer based on silicon substrate as the bottom cladding layer and then baked at 120 °C for 2 h. On the cladding layer, the standard pattern exposure was performed at a wavelength of 365 nm using the 350 mW Hg lamp through a contact chromium mask. And ICP etching technique was operated using oxygen. Then the NaYF_4_/NaLuF_4_:Yb^3+^, Er^3+^ NPs-PMMA was embedded into the grooves to form the core waveguides via spin-coating, and then the device was baked at 120 °C for 2 h. Finally, a 3-μm-thick PMMA film was spin-coated as the upper cladding and then baked at 120 °C for 2 h.

### Measurement setup

**Figure 6:**
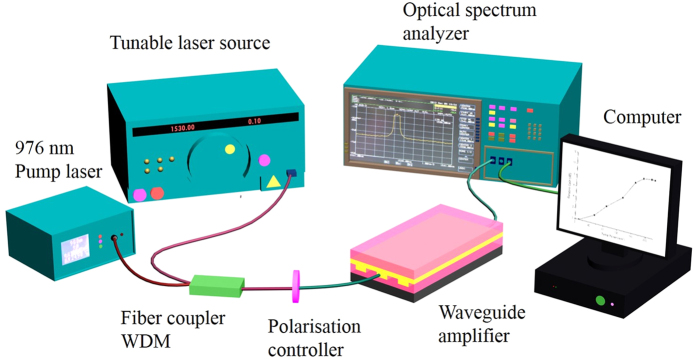
The schematic of the experimental setup for the optical gain measurement.

Figure 6 shows a schematic of the experimental setup for the optical gain measurement. The relative gain of the waveguide amplifiers was performed by using a tunable laser source (Santec TSL-210) with working wavelength range of 1510 nm to 1590 nm as the signal source and a 976 nm continuous wave laser diode as the pump source, with an output power of 400 mW. Signal and pump light were launched into the channel waveguides by a 976/1535 nm wavelength division multiplexing (WDM) coupler. A polarization controller was used to guarantee the input light was operated under TM mode. The output light from the device was collected and coupled to an optical spectrum analyzer (OSA, Ando AQ-6315A). Next, the relative gain was calculated via a computer using formula 14, where *P*s(*z*) is the output signal power amplified by the waveguide, and *P*s(0) is the input signal power.

## Additional Information

**How to cite this article**: Zhang, M.L. *et al.* High-gain polymer optical waveguide amplifiers based on core-shell NaYF_4_/NaLuF_4_: Yb^3+^, Er^3+^ NPs-PMMA covalent-linking nanocomposites. *Sci. Rep.* **6**, 36729; doi: 10.1038/srep36729 (2016).

**Publisher’s note**: Springer Nature remains neutral with regard to jurisdictional claims in published maps and institutional affiliations.

## Figures and Tables

**Table 1:** Parameters of NaYF_4_/NaLuF_4_: 20% Yb^3+^, 2% Er^3+^ NPs-PMMA covalent-linking nanocomposite waveguide amplifier.

Total Er^3+^ concentration	*N*_Er_ = 2.8 × 10^26^ m^−3^
Total Yb^3+^ concentration	*N*_Yb_ = 10 *N*_Er_
Er^3+^ absorption cross-section	σ_12_ = 9.5 × 10^−24^ m^2^
Er^3+^ emission cross-section	σ_21_ = 2.7 × 10^−24^ m^2^
Er^3+^ absorption cross-section	σ_13_ = 2.36 × 10^−25^ m^2^
Yb^3+^ absorption cross-section	σ^Yb^_56_ = 1.0 × 10^−23^ m^2^
Yb^3+^ emission cross-section	σ^Yb^_65_ = 1.0 × 10^−23^ m^2^
Er^3+^ emission lifetime on level ^4^I_13/2_	τ_21_ = 12 ms
Er^3+^ non-radiation lifetime on level ^4^I_11/2_	τ_32_ = 0.38 ms
Yb^3+^ emission lifetime on level ^2^F_5/2_	τ^Yb^_65_ = 1.86 ms
Er^3+^ upconversion coefficient	*C*_up_ = 4.1 × 10^−23^ m^3^s^−1^
Er^3+^ cross-relaxation coefficient	*C*_Cr_ = 3.4 × 10^−22^ m^3^s^−1^
Overlapping factor of the signal	Γs = 0.848
Overlapping factor of the pump	Γp = 0.87
Signal power	Ps = 0.1 mW
Pump power	Pp = 400 mW
Waveguide length	L = 1.3 cm
